# Synthesis and Anticancer Activity of New Thiopyrano[2,3-*d*]thiazoles Based on Cinnamic Acid Amides

**DOI:** 10.3797/scipharm.1408-05

**Published:** 2014-09-15

**Authors:** Andrii Lozynskyi, Borys Zimenkovsky, Roman Lesyk

**Affiliations:** Department of Pharmaceutical, Organic and Bioorganic Chemistry, Danylo Halytsky Lviv National Medical University, Pekarska 69, 79010, Lviv, Ukraine.

**Keywords:** hetero-Diels-Alder reaction, Cynnamic acid amides, 5-Ylideneisorhodanines, Thiopyrano[2,3-d][1,3]thiazoles, Anticancer activity

## Abstract

Novel rel-(5R,6S,7S)-2-oxo-5-phenyl-7-aryl(hetaryl)-3,7-dihydro-2H-thiopyrano [2,3-d]thiazole-6-carboxylic acid amides were synthesized in a hetero-Diels-Alder reaction with a series of cinnamic acid amides. The synthesized compounds were tested for their anticancer activity in vitro in the standard National Cancer Institute 60 cancer cell line assay. Promising compounds 3e, 3g, and 3h with moderate antitumor activity were identified among the synthesized series.

## Introduction

Investigations of thiopyrano[2,3-*d*]thiazole derivatives, the isosteric mimics of biologically active 5-ylidene-4-thiazolidinones, led to the synthesis of compounds with anticancer, antitrypanosomal, and antimycobacterial properties which can provide an opportunity to further study and explore the pharmacological activity of these heterocyclic systems in the future [1–13]. We decided to combine in a single heterocyclic system the thiazolidinone moiety and a fragment of cinnamic acid (Sch. 1). Cinnamic acid and its derivatives exhibit antitumor, antimicrobial, antifungal action and act as histamine H_3_-receptor antagonists [14–16]. Consequently, we have synthesized thiopyrano[2,3-*d*]thiazoles using cinnamic acid amides as the dienophile in the reaction of *hetero*-Diels-Alder.

In addition, heterodiene synthesis allows the fixing of the biologically important 4-thiazolidinone fragment in a rigid fused system, preserving its biological activity. Moreover, the combination of thiazole and thiopyran in a fused heterosystem is a precondition for creating ligand-target binding and enhances the potential selectivity to biotargets. This approach suggests the critical impact of the substituent on the biological activity with particular selectivity to various cancer cell lines.

**Sch. 1.**
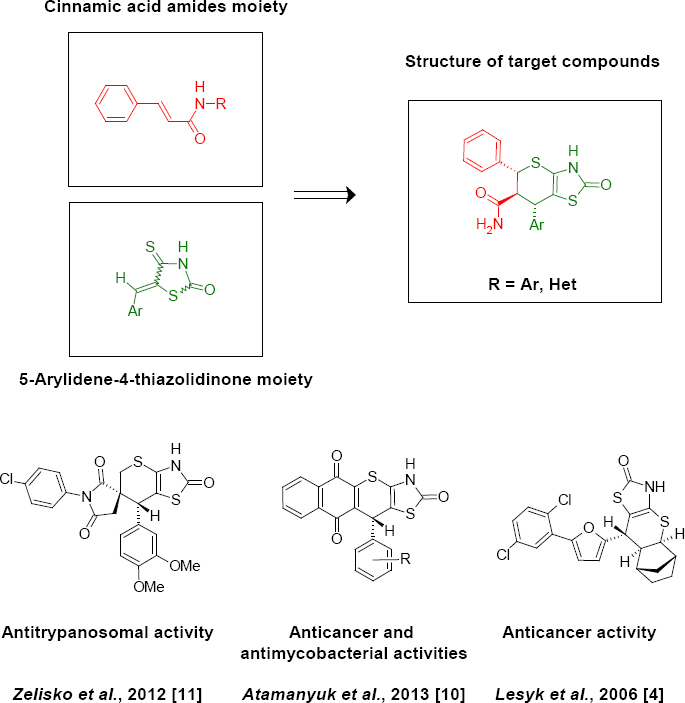
Background for the synthesis of target compounds

## Results and Discussion

### Chemistry

The starting 5-aryl(hetaryl)idene-4-thioxo-2-thiazolidinones **1a–d** were obtained by the treatment of 4-thioxo-2-thiazolidinone with the appropriate aldehydes in glacial acetic acid with a catalytic amount of fused sodium acetate [4, 12]. The cinnamic acid amides were synthesized by the interaction of the corresponding cinnamic acid chloride with 4-substituted anilines, morpholine, and 2-aminopyridine in anhydrous dioxane. The *hetero*-Diels-Alder reaction of **2a–f** with 5-aryl(hetaryl)idene-4-thioxo-2-thiazolidinones **1a–d** yielded a series of novel *rel*-(5*R*,6*S*,7*S*)-2-oxo-5-phenyl-7-aryl(hetaryl)-3,7-dihydro-*2H*-thiopyrano[2,3-*d*]thiazole-6-carboxylic acid amides (Sch. 2).

**Sch. 2.**
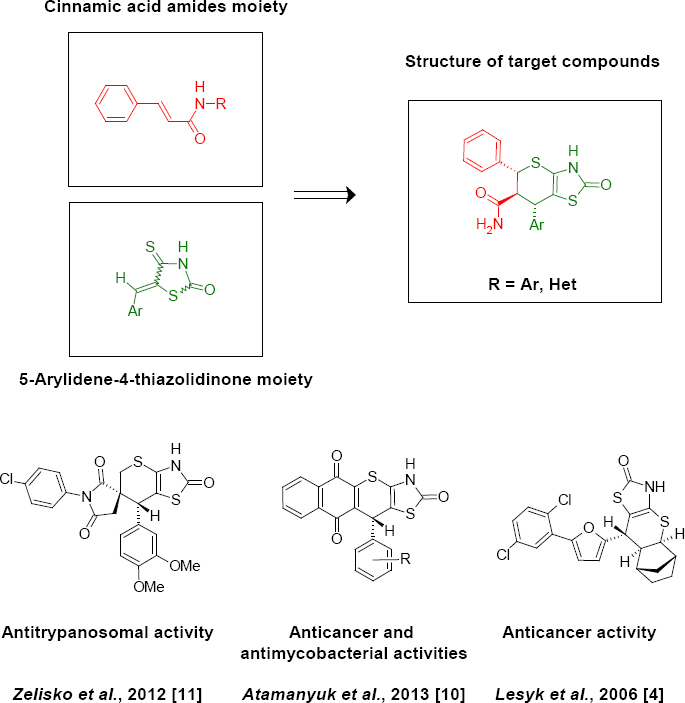
Synthesis of 2-oxo-5-phenyl-7-aryl(hetaryl)-3,7-dihydro-2*H*-thiopyrano[2,3-*d*]-thiazole-6-carboxylic acid amides

The structure of the synthesized compounds was confirmed by ^1^H- and ^13^C NMR. We found the features of the stereochemistry of the above *hetero*-Diels-Alder reaction. Particularly, we have observed that cinnamic acid amides in the [4+2]-cyclocondensation of 5-arylideneisorhodanines form a pair of *rel*-(5*R*,6*S*,7*S*)-diastereomers. This claim is based on the coupling constant values within 10.4–11.5 Hz and the spectral signals of the thiopyran fragment (triplet and two doublets at 3.40–4.87 ppm), which prove an axial-axial interaction of 5-H, 6-H and 6-H, 7-H proton pairs. Importantly, a similar pattern was observed earlier for cinnamic acids as the dienophile in the reactions of *hetero*-Diels-Alder [11, 12].

**Tab. 1.** Cytotoxic activity of the tested compounds in the concentration 10^-5^ M against 60 cancer cell lines

Test cpds.	Average growth, %	Range of growth, %	Most sensitive cell line growth, % (cancer line/type)
3b	82.76	53.05–106.26	53.05 (*RPMI-8226* / leukemia)
			61.05 (*SF-295* / CNS cancer)
3c	101.41	88.61–117.37	88.61 (*RXF 393* / renal cancer)
**3e**	**57.09**	**26.38–94.10**	**27.11 (MOLT–4 / leukemia)**
			**26.38 (HCT–116 / colon cancer)**
			**32.89 (SF–295 / CNS cancer)**
			**35.53 (PC–3 / prostate cancer)**
			**33.44 (MCF7 / breast cancer)**
			**33.81 (*T–47D* / breast cancer)**
**3g**	**57.89**	**26.51–91.71**	**26.51 (*MOLT–4* / leukemia);**
			**37.02 (*RPMI–8226* / leukemia)**
			**39.39 (*A549/ATCC* / non-small cell lung cancer)**
			**32.09 (*HCT–116* / colon cancer)**
			**33.18 (*SF–295* / CNS cancer)**
			**33.99 (*PC–3* / prostate cancer)**
			**31.77 (*MCF7* / breast cancer)**
			**39.88 (*T–47D* / breast camcer)**
**3h**	**77.68**	**–42.92–114.10**	**30.79 (*HOP–92* / non-small cell lung cancer)**
			**-42.92 (*NCI–H522* / non-small cell lung cancer)**
			**35.63 (*SK-MEL-5* / melanoma)**
			**-21.73 (*CAKI-1* / renal cancer)**
			**37.39 (*UO-31* / renal cancer)**
3i	80.64	51.43–119.84	57.75 (*SF-295* / CNS cancer)
			51.43 (*PC-3* / prostate cancer)
			59.03 (*MCF7* / breast cancer)
3j	88.76	61.65–112.27	61.65 (*SNB-75* / CNS cancer)
3k	95.84	72.63–120.48	72.63 (*T-47D* / breast cancer)
3l	100.81	73.58–120.80	77.27 (*SNB-75* / CNS cancer)
			78.29 (*UO-31* / renal cancer)
			73.58 (*T-47D* / breast cancer)
3m	96.07	73.69–110.93	73.69 (*SR* / leukemia);

### Biological Activity

The synthesized *rel*-(5*R*,6*S*,7*S*)-2-oxo-5-phenyl-7-aryl(hetaryl)-3,7-dihydro-*2H*-thiopyrano-[2,3-*d*]thiazole-6-carboxylic acid amides were selected by the National Cancer Institute (NCI) Developmental Therapeutic Program (www.dtp.nci.nih.gov) for the *in vitro* cell line screening to investigate their anticancer activity. Anticancer assays were performed according to the NCI protocol, which is described elsewhere [5–7, 17]. The compounds were evaluated for antitumor activity against 60 cancer lines at a 10 µM concentration. The human tumor cell lines were derived from nine different cancer types: leukemia, melanoma, lung, colon, CNS, ovarian, renal, prostate, and breast cancers. The screening results are shown in Table 1.

The tested compounds showed different levels of activity on various cancer cell lines. The most active compounds were **3e**, **3g**, **3h**, being highly potent in certain lines of cancer, but they had almost no activity in others. Compounds **3e**, **3g** have a selective effect on the growth of MOLT-4 (leukemia), HCT-116 (colon cancer), SF-295 (CNS cancer), PC-3 (prostate cancer), MCF7, and T-47D (breast cancer) cancer cell lines in comparison with others.

**Sch. 3.**
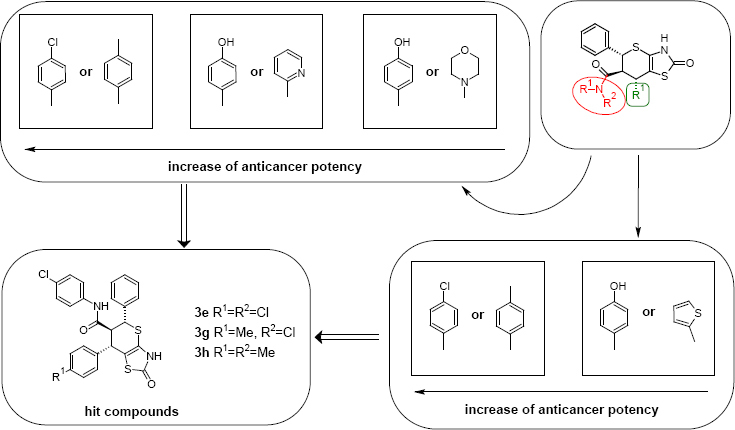
SAR of anticancer potency of the synthesized thiopyrano[2,3-*d*]thiazole-6-carboxylic acids amides

The empirical SAR study (Sch. 3) revealed that:

(1) the anticancer activity of the synthesized compounds is sensitive to the nature of the amide fragment in position 6 and substitution in position 7 of the thiopyrano[2,3-*d*]thiazole moiety;

(2) introduction of *p*-Me- or *p*-Cl-C6H4 groups in the amide fragment enhances the potency;

(3) the loss of anticancer activity is caused by the introduction of morpholin and pyridine fragments in position 6 or substitution of the arylamide moiety by OH or sulfanilamido groups;

(4) synthesized thiopyrano[2,3-d]thiazole-6-carboxylic acid amides with *p*-Me- and *p*-Cl-C6H4 groups in postion 7 have the most preferable level of activity compared to other derivatives.

## Experimental

### Chemistry

All materials were purchased from Merck, Sigma-Aldrich, or Lancaster and were used without purification. 5-Aryl(hetaryl)idene-4-thioxo-2-thiazolidinones **1a-d** were employed as starting materials and prepared according to the method described previously [4, 12]. Melting points were determined in open capillary tubes and were uncorrected. The elemental analyses (C, H, N) were performed using the Perkin—Elmer 2400 CHN analyzer and were within 0.4% of the theoretical values. The ^1^H- and ^13^C NMR spectra were recorded on the Varian Gemini 400 MHz or Bruker 125 MHz for frequencies of 100 MHz in DMSO-*d*_6_ using tetramethylsilane as an internal standard. Chemical shifts are reported in ppm units with the use of a δ scale. The purity of all obtained compounds was checked by ^1^H-NMR and TLC.

### General Procedure of the Hetero-Diels-Alder Reaction Affording 3a-m

A mixture of appropriate 5-aryl(hetaryl)idene-4-thioxo-2-thiazolidinone (5 mmol) and cinnamic acid amide (5.5 mmol) was refluxed for 4–7 h with a catalytic amount of hydroquinone (2–3 mg) in 15 ml of glacial acetic acid and left overnight at room temperature. The obtained solid products were collected by filtration, washed with water, methanol (5–10 ml), diethyl ether, and recrystallized from the appropriate solvent.

rel-(5R,6S,7S)-N-(4-Chlorophenyl)-7-(4-hydroxyphenyl)-2-oxo-5-phenyl-3,5,6,7-tetrahydro-2H-thiopyrano[2,3-d][1,3]thiazole-6-carboxamide (**3a**)

Yield: 59%, mp 234–236°C (EtOH). ^1^H NMR (400 MHz, DMSO-*d*_6_) **δ**: 3.47 (t, 1H, *J =* 10.4 Hz, 6-H), 4.21 (d, 1H, *J =* 10.4 Hz, 7-H), 4.83 (d, 1H, *J =* 10.4 Hz, 5-H), 6.70 (d, 2H, *J =* 8.8 Hz, arom.), 7.00 (d, 2H, *J =* 8.8 Hz, arom.), 7.12 (d, 2H, *J =* 8.8 Hz, arom.), 7.20 (t, 1H, *J =* 7.2 Hz, arom.), 7.28 (t, 2H, *J =* 7.2 Hz, arom.), 7,46 (d, 2H, *J =* 7.2 Hz, arom.), 9,38 (s, 1H, OH), 10.27 (s, 1H, NH), 11.50 (s, 1H, NH). ^13^C NMR (100 MHz, DMSO-*d*_6_) **δ**: 170.7, 167.3, 156.6, 138.4, 137.3, 136.6, 130.3, 129.6, 128.5, 128.3, 128.2, 128.0, 127.1, 126.7, 121.0, 120.5, 56.3, 51.1, 42.1. Anal. Calcd for C_25_H_19_CIN_2_O_3_S_2_, % C, 60.66; H, 3.87; N, 5.66. Found, %: C, 60.80; H, 3.80; N, 5.80.

rel-(5R,6S,7S)-7-(4-Chlorophenyl)-N-(4-hydroxyphenyl)-2-oxo-5-phenyl-3,5,6,7-tetrahydro-2H-thiopyrano[2,3-d][1,3]thiazole-6-carboxamide (**3b**)

Yield: 57%, mp 220–224°C (EtOH). ^1^H NMR (400 MHz, DMSO-*d*_6_) **δ**: 3.44 (t, 1H, *J =* 10.4 Hz, 6-H), 4.28 (d, 1H, *J =* 10.4 Hz, 7-H), 4.68 (d, 1H, *J =* 10.4 Hz, 5-H), 6.47 (d, 2H, *J =* 8.8 Hz, arom.), 6.60 (d, 2H, *J =* 8.8 Hz, arom.), 7.00–7.40 (m, 7H, arom.), 7.50 (d, 2H, *J =* 7.2 Hz, arom.), 9,20 (s, 1H, OH), 9.83 (s, 1H, NH), 11.53 (s, 1H, NH). ^13^C NMR (100 MHz, DMSO-*d*_6_) **δ**: 170.6, 164.5, 140.2, 139.4, 138.8, 136.3, 129.2, 128.9, 128.6, 128.3, 128.2, 128.1, 127.8, 126.9, 121.6, 114.8, 51.9, 50.8, 46.7. Anal. Calcd for C_25_H_19_CIN_2_O_3_S_2_, % C, 60.66; H, 3.87; N, 5.64. Found, %: C, 60.50; H, 3.70; N, 5.70.

rel-(5R,6S,7S)-7-(4-Methylphenyl)-2-oxo-5-phenyl-N-(4-sulfamoylphenyl)-3,5,6,7-tetrahydro-2H-thiopyrano[2,3-d][1,3]thiazole-6-carboxamide (**3c**)

Yield: 60%, mp 188–190°C (EtOH). ^1^H NMR (400 MHz, DMSO-*d*_6_) **δ**: 2.27 (s, 3H, CH_3_), 3.95 (t, 1H, *J =* 11.4 Hz, 6-H), 4.47 (d, 1**H**, *J =* 11.4 Hz, 7-H), 4.73 (d, 1**H**, *J =* 11.4 Hz, 5-H), 6.98 (d, 2H, *J* = 7.8 Hz, arom.), 7.09 (d, 2H, *J =* 7.8 Hz, arom.), 7.28 (s, 2H, NH2), 7.30–7.50 (m, 5H, arom.), 7.80 (d, 2H, *J =* 9.0 Hz, arom.), 7.87 (d, 2H, *J =* 9.0 Hz, arom.), 10.49 (s, 1H, NH), 11.41 (s, 1H, NH). ^13^C NMR (100 MHz, DMSO-*d*_6_) **δ**: 167.6, 163.8, 142.0, 140.9, 138.3, 136.6, 134.4, 129.9, 129.0, 127.7, 126.7, 121.7, 120.5, 118.7, 118.4, 104.8, 51.1, 42.3, 42.1, 20.7. Anal. Calcd for C_26_H_23_N_3_O_4_S_3_, % C, 58.08; H, 4.31; N, 7.82. Found, %: C, 58.20; H, 4.40; N, 7.80.

rel-(5R,6S,7S)-7-(4-Chlorophenyl)-2-oxo-5-phenyl-N-(4-sulfamoylphenyl)-3,5,6,7-tetrahydro-2H-thiopyrano[2,3-d][1,3]thiazole-6-carboxamide (**3d**)

Yield: 68%, mp 182–184°C (EtOH). ^1^H NMR (400 MHz, DMSO-*d*_6_) **δ**: 3.95 (t, 1H, *J =* 11.6 Hz, 6-H), 4.46 (d, 1**H**, *J =* 11.6 Hz, 7-H), 4.65 (d, 1**H**, *J =* 11.6 Hz, 5-H), 7.08 (d, 2H, *J =* 8.8 Hz, arom.), 7.27 (d, 2H, *J =* 8.8 Hz, arom.), 7.29 (s, 2H, NH2), 7.30–7.50 (m, 5H, arom.), 7.76 (d, 2H, *J =* 8.6 Hz, arom.), 7.83 (d, 2H, *J =* 8.6 Hz, arom.), 10.56 (s, 1H, NH), 11.55 (s, 1H, NH). ^13^C NMR (100 MHz, DMSO-*d*_6_) **δ**: 171.0, 167.6, 163.8, 142.0, 140.9, 138.3, 130.5, 129.9, 128.9, 128.4, 127.7, 126.6, 126.5, 121.7, 118.5, 104.1, 56.0, 51.0, 42.1. Anal. Calcd for C_25_H_20_CIN_3_O_4_S_3_, % C, 53.80; H, 3.61; N, 7.53. Found, %: C, 53.70; H, 3.80; N, 7.40.

rel-(5R,6S,7S)-N,7-Bis(4-chlorophenyl)-2-oxo-5-phenyl-3,5,6,7-tetrahydro-2H-thiopyrano[2,3-d][1,3]thiazole-6-carboxamide (**3e**)

Yield: 65%, mp 216–218°C (EtOH). ^1^H NMR (400 MHz, DMSO-*d*_6_) **δ**: 3.44 (t, 1H, *J =* 10.4 Hz, 6-H), 4.31 (d, 1**H**, *J =* 10.4 Hz, 7-H), 4.72 (d, *J =* 10.4 Hz, 5-H), 6.96 (d, 2H, *J =* 8.4 Hz, arom.), 7.03 (d, 2H, *J =* 8.4 Hz, arom.), 7.16–7.31 (m, 7H, arom.), 7.44 (d, 2H, *J =* 7.0 Hz, arom.), 9.46 (s, 1H, NH), 11.31 (s, 1H, NH). ^13^C NMR (100 MHz, DMSO-*d*_6_) **δ**: 170.4, 168.3, 139.2, 136.4, 136.1, 132.1, 131.1, 130.1, 128.5, 128.4, 128.3, 128.2, 127.3, 121.0, 120.3, 107.4, 56.1, 48.4, 44.9. Anal. Calcd for C_25_H_18_CI_2_N_2_O_2_S_2_, % C, 58.48; H, 3.53; N, 5.46. Found, %: C, 58.30; H, 3.40; N, 5.50.

rel-(5R,6S,7S)-N-(4-Chlorophenyl)-7-(4-methylphenyl)-2-oxo-5-phenyl-3,5,6,7-tetrahydro-2H-thiopyrano[2,3-d][1,3]thiazole-6-carboxamide (**3f**)

Yield: 75%, mp 200–202°C (EtOH). ^1^H NMR (400 MHz, DMSO-c/6) **δ**: 2.28 (s, 3H, CH_3_), 3.44 (t, 1H, *J =* 10.4 Hz, 6-H), 4.26 (d, 1H, *J =* 10.4 Hz, 7-H), 4.70 (d, 1H, *J =* 10.4 Hz, 5-H), 6.96 (d, 2H, *J =* 8.8 Hz, arom.), 7.02 (d, 2H, *J =* 8.8 Hz, arom.), 7.05 (d, 2H, *J =* 7.6 Hz, arom.), 7.13 (d, 2H, *J =* 7.6 Hz, arom.), 7.18 (t, 1H, *J =* 7.2 Hz, arom.), 7.24 (t, 2H, *J =* 7.2 Hz, arom.), 7.44 (d, 2H, *J =* 7.2 Hz, arom.), 9.41 (s, 1H, NH), 11.23 (s, 1H, NH). ^13^C NMR (100 MHz, DMSO-*d*_6_) **δ**: 170.5, 168.5, 137.2, 136.7, 136.5, 136.2, 129.0, 128.5, 128.3, 128.2, 128.1, 127.1, 119.8, 108.3, 56.2, 48.6, 45.2, 20.7. Anal. Calcd for C_26_H_21_CIN_2_O_2_S_2_, % C, 63.34; H, 4.29; N, 5.68. Found, %: C, 63.50; H, 4.40; N, 5.70.

rel-(5R,6S,7S)-7-(4-Chlorophenyl)-N-(4-methylphenyl)-2-oxo-5-phenyl-3,5,6,7-tetrahydro-2H-thiopyrano[2,3-d][1,3]thiazole-6-carboxamide (**3g**)

Yield: 70%, mp 234–236°C (EtOH). ^1^H NMR (400 MHz, DMSO-*d*_6_) **δ**: 2.16 (s, 3H, CH_3_), 3.44 (t, 1H, *J =* 10.4 Hz, 6-H), 4.30 (d, 1H, *J =* 10.4 Hz, 7-H), 4.70 (d, 1H, *J =* 10.4 Hz, 5-H), 6.75 (d, 2H, *J =* 8.4 Hz, arom.), 6.83 (d, 2H, *J =* 8.4 Hz, arom.), 7.20–7.30 (m, 7H, arom.), 7.46 (d, 2H, *J =* 7.2 Hz, arom.), 9.23 (s, 1H, NH), 11.29 (s, 1H, NH). ^13^C NMR (100 MHz, DMSO-*d*_6_) **δ**: 170.4, 167.9, 139.3, 136.2, 134.9, 132.7, 132.0, 130.2, 128.6, 128.5, 128.4, 128.3, 120.4, 119.8, 119.1, 107.5, 55.8, 48.5, 45.0, 20.3. Anal. Calcd for C_26_H_21_CIN_2_O_2_S_2_, % C, 63.34; H, 4.29; N, 5.68. Found, %: C, 63.20; H, 4.40; N, 5.70.

rel-(5R,6S,7S)-N,7-Bis(4-methylphenyl)-2-oxo-5-phenyl-3,5,6,7-tetrahydro-2H-thiopyrano[2,3-d][1,3]thiazole-6-carboxamide (**3h**)

Yield: 56%, mp 230–232°C (EtOH). ^1^H NMR (400 MHz, DMSO-*d*_6_) **δ**: 2.16 (s, 3H, CH_3_), 2.28 (s, 3H, CH_3_), 3.45 (t, 1H, *J =* 10.4 Hz, 6-H), 4.26 (d, 1H, *J =* 10.4 Hz, 7-H), 4.70 (d, 1H, *J =* 10.4 Hz, 5-H), 6.77 (d, 2H, *J =* 8.0 Hz, arom.), 6.82 (d, 2H, *J =* 8.0 Hz, arom.), 7.06 (d, 2H, *J =* 7.6 Hz, arom.), 7.14 (d, 2H, *J =* 7.6 Hz, arom.), 7.21 (t, 1H, *J =* 7.2 Hz, arom.), 7.25 (t, 2H, *J =* 7.2 Hz, arom.), 7.46 (d, 2H, *J =* 7.2 Hz, arom.), 9.18 (s, 1H, NH), 11,22 (s, 1H, NH). ^13^C NMR (100 MHz, DMSO-*d*_6_) **δ**: 170.5, 168.1, 141.2, 140.1, 137.4, 136.6, 136.4, 135.1, 132.5, 128.9, 128.6, 128.2, 128.1, 119.8, 119.7, 108.5, 55.9, 48.7, 45.2, 20.7, 20.3. Anal. Calcd for C_27_H_24_N_2_O_2_S_2_, % C, 68.62; H, 5.12; N, 5.93. Found, %: C, 68.70; H, 5.20; N, 6.00.

rel-(5R, 6S,7S)-N-(4-Chlorophenyl)-2-oxo-5-phenyl-7-(thiophen-2-yl)-3,5,6,7-tetrahydro-2H-thiopyrano[2,3-d][1,3]thiazole-6-carboxamide (**3i**)

Yield: 84%, mp 208–210°C (EtOH). ^1^H NMR (400 MHz, DMSO-*d*_6_) **δ**: 3.55 (t, 1H, *J =* 10.5 Hz, 6-H), 4.72 (d, 1H, *J =* 10.5 Hz, 7-H), 4.87 (d, *J =* 10.5 Hz, 5-H), 6.92 (dd, 1H, *J =* 5.1, 3.6 Hz, thiophen.), 6.98 (d, 1**H**, *J =* 2.4 Hz, thiophen.), 7.05 (d, 2H, *J =* 9.0 Hz, arom.), 7.13 (d, 2H, *J =* 7.6 Hz, arom.), 7.17 (d, 2H, *J =* 8.4 Hz, arom.), 7.26 (t, 1H, *J =* 7.0 Hz, arom.), 7.30 (t, 2H, *J =* 7.5 Hz, arom.), 7.45 (d, 1**H**, *J =* 5.1 Hz, thiophen.), 7.51 (d, 2H, *J =* 7.2 Hz, arom.), 9.46 (s, 1H, NH), 11.31 (s, 1H, NH). ^13^C NMR (100 MHz, DMSO-*d*_6_) **δ**: 170.3, 168.3, 142.8, 136.5, 136.0, 128.5, 128.4, 128.3, 128.2, 127.2, 126.7, 126.6, 125.8, 121.0, 119.9, 107.8, 59.6, 56.8, 48.6. Anal. Calcd for C_24_H_24_N_2_O_3_S_2_, % C, 56.95; H, 3.53; N, 5.78. Found, %: C, 56.80; H, 3.70; N, 5.60.

rel-(5R,6S,7S)-7-(4-Chlorophenyl)-2-oxo-5-phenyl-N-(pyridin-2-yl)-3,5,6,7-tetrahydro-2H-thiopyrano[2,3-d][1,3]thiazole-6-carboxamide (**3j**)

Yield: 56%, mp 178–180°C (AcOH). ^1^H NMR (400 MHz, DMSO-*d*_6_) **δ**: 3.48 (t, 1H, *J =* 10.5 Hz, 6-H), 4.24 (d, 1H, *J =* 10.5 Hz, 7-H), 4.84 (d, *J =* 10.5 Hz, 5-H), 7.16–7.45 (m, 9H, arom., pyrid.), 10.21 (s, 1H, NH), 11.50 (s, 1H, NH). ^13^C NMR (100 MHz, DMSO-*d*_6_) **δ**: 171.7, 169.5, 150.6, 147.5, 139.2, 137.8, 136.1, 132.1, 130.3, 128.7, 128.5, 128.4, 128.3, 120.3, 119.4, 113.0, 107.5, 54.4, 48.6, 45.4. Anal. Calcd for C_24_H_18_CIN_3_O_2_S_2_, % C, 60.05; H, 3.78; N, 8.75. Found, %: C, 60.10; H, 3.70; N, 8.90.

rel-(5R,6S,7S)-2-Oxo-5-phenyl-N-(pyridin-2-yl)-7-(thiophen-2-yl)-3,5,6,7-tetrahydro-2H-thiopyrano[2,3-d][1,3]thiazole-6-carboxamide (**3k**)

Yield: 76%, mp 150–152°C (AcOH). ^1^H NMR (400 MHz, DMSO-*d*_6_) **δ**: 3.43 (t, 1H, *J =* 10.5 Hz, 6-H), 4.62 (d, 1H, *J =* 10.5 Hz, 7-H), 4.84 (d, *J =* 10.5 Hz, 5-H), 7.20–7.61 (m, 9H, arom., thiophen., pyrid.), 7.86 (d, 1H, *J =* 4.0 Hz, thiophen.), 8.10–8.20 (m, 2H, pyrid.), 10.29 (s, 1H, NH), 11.47 (s, 1H, NH). ^13^C NMR (100 MHz, DMSO-*d*_6_) **δ**: 171.8, 170.4, 150.8, 147.5, 142.5, 137.7, 136.1, 134.6, 129.3, 128.7, 128.5, 128.3, 126.6, 125.9, 119.8, 113.1, 107.9, 56.1, 48.8, 47.9. Anal. Calcd for C_22_H_17_N_3_O_2_S_3_, % C, 58.51; H, 3.79; N, 9.30. Found, %: C, 58.40; H, 3.90; N, 9.20.

rel-(5R,6S,7S)-7-(4-Chlorophenyl)-6-(morpholin-4-ylcarbonyl)-5-phenyl-3,5,6,7-tetrahydro-2H-thiopyrano[2,3-d][1,3]thiazol-2-one (**3l**)

Yield: 90%, mp 206–208°C (EtOH). ^1^H NMR (400 MHz, DMSO-*d*_6_) **δ**: 3.44 (t, 1H, *J =* 10.4 Hz, 6-H), 3.45–3.55 (m, 4H, morpholin), 3.73–3.81 (m, 2H, morpholin), 4.24 (d, 1H, *J =* 10.4 Hz, 7-H), 4.64 (d, 1H, *J =* 10.4 Hz, 5-H), 7.10 (d, 2H, *J =* 8.0 Hz, arom.), 7.18–7.34 (m, 7H, arom.), 11.33 (s, 1H, NH). ^13^C NMR (100 MHz, DMSO-*d*_6_) **δ**: 170.7, 166.8, 138.8, 131.8, 131.0, 128.2, 128.0, 129.9, 127.9, 127.8, 120.9, 104.6, 66.5, 66.1, 56.0, 45.3, 42.9, 41.4. Anal. Calcd for C_23_H_21_ClN_2_O_3_S_2_, % C, 58.40; H, 4.47; N, 5.92. Found, %: C, 58.50; H, 4.30; N, 6.00.

rel-(5R,6S,7S)-7-(4-Methylphenyl)-6-(morpholin-4-ylcarbonyl)-5-phenyl-3,5,6,7-tetrahydro-2H-thiopyrano[2,3-d][1,3]thiazol-2-one (**3m**)

Yield: 77%, mp 176–178°C (PhH). ^1^H NMR (400 MHz, DMSO-*d*_6_) **δ**: 2.33 (s, 3H, CH_3_), 2.77–2.86 (m, 4H, morpholin), 2.92–2.95 (m, 2H, morpholin), 3.76 (T, 1H, *J =* 10.4 Hz, 6-H), 4.14 (d, 1H, *J =* 10.4 Hz, 7-H), 4.69 (d, 1H, *J =* 10.4 Hz, 5-H), 7.10 (br.s, 4H, arom.), 7.25–7.34 (m, 3H, arom.), 7.40 (d, 2H, *J =* 7.0 Hz, arom.), 11.25 (s, 1H, NH). ^13^C NMR (100 MHz, DMSO-*d*_6_) **δ**: 170.5, 168.7, 146.3, 145.3, 136.9, 136.8, 128.9, 128.5, 128.3, 128.2, 120.1, 65.6, 49.2, 48.7, 45.7, 45.3, 41.3, 20.9. Anal. Calcd for C_24_H_24_N_2_O_3_S_2_, % C, 58.40; H, 4.47; N, 5.92. Found, %: C, 58.30; H, 4.50; N, 5.80.

### Cytotoxic Activity Against Malignant Human Tumor Cells

An anticancer *in vitro* assay was performed on the human tumor cell lines panel derived from nine neoplastic diseases, in accordance with the protocol of the Drug Evaluation Branch, National Cancer Institute, Bethesda [5–7, 17]. The tested compounds were added to the culture at a single concentration (10^-5^ M) and the cultures were incubated for 48 h. Endpoint determinations were made with a protein binding dye, sulforhodamine B (SRB). Results for each tested compound were reported as the percent of growth of the treated cells when compared to the untreated control cells. The growth percentage was evaluated spectrophotometrically versus controls not treated with the test agents.
